# Report of a Rare Case of Focal Depressed Fracture of the Tibia in an Adult

**DOI:** 10.7759/cureus.22332

**Published:** 2022-02-17

**Authors:** Parag S Mahajan, Jouhar J Kolleri, Sakshi Prasad, El Habib Belhaddad, Sarah Ait Souabni, Hussain Mohammed

**Affiliations:** 1 Clinical Imaging Department, Hamad Medical Corporation, Doha, QAT; 2 Internal Medicine, National Pirogov Memorial Medical University, Vinnytsya, UKR; 3 Family Medicine, Cadi Ayyad University, Faculty of Medicine and Pharmacy of Marrakesh, Marrakech, MAR; 4 Surgery, Hamad Medical Corporation, Doha, QAT

**Keywords:** magnetic resonsonance imaging, computed tomography, tibial depressed fracture, depressed fracture, fracture

## Abstract

Focal depressed fracture of the proximal tibial metaphysis without any articular involvement is a rare condition. We present a case of a 46-year-old man with a lacerated wound over the right proximal tibia after a history of slip and fall. The imaging involved to diagnose the condition included an X-ray, computed tomography (CT), and magnetic resonance imaging (MRI) to exclude a pathological fracture and to better delineate the relationship between the fractured bone and the surrounding structures. Conservative management was indicated and implemented with a positive outcome.

## Introduction

Depressed fractures of the tibial plateau are common and are caused by high-energy trauma [1]. The case we are presenting involves an unusual focal depression fracture of the proximal tibial metaphysis without any articular involvement and without any underlying bone pathology. A 46-year-old man presented with excessive pain on palpation of the right leg and inability to walk after a history of slip and fall and severe trauma to the proximal part of the right leg. Local examination showed the presence of a lacerated wound over the right proximal tibia. On imaging, a focal depressed fracture was noted involving the anteromedial cortex of the right proximal tibia. The depressed fracture of the upper extremity of the tibia without the involvement of the articular surface and without any underlying bone pathology is not reported in the literature to date, to the best of our knowledge. Fractures of the proximal tibia are challenging to manage as the bone is a load-bearing one that creates difficulties during consolidation and may cause malunions. The anterior border of the shaft lacks protection with soft tissue which also reduces blood supply and slows down the regenerative process [2]. 

## Case presentation

A 46-year-old gentleman presented to the emergency department with a history of slip and fall and complained of right knee pain. On examination, he was conscious and oriented. Vitals and basic laboratory investigations were within normal limits. On local examination, there was a lacerated wound over the right proximal tibia measuring 1 x 2 cm. Hip and ankles were stable and there were no neurovascular deficits. Other systemic examinations were within normal limits.

**Figure 1:**
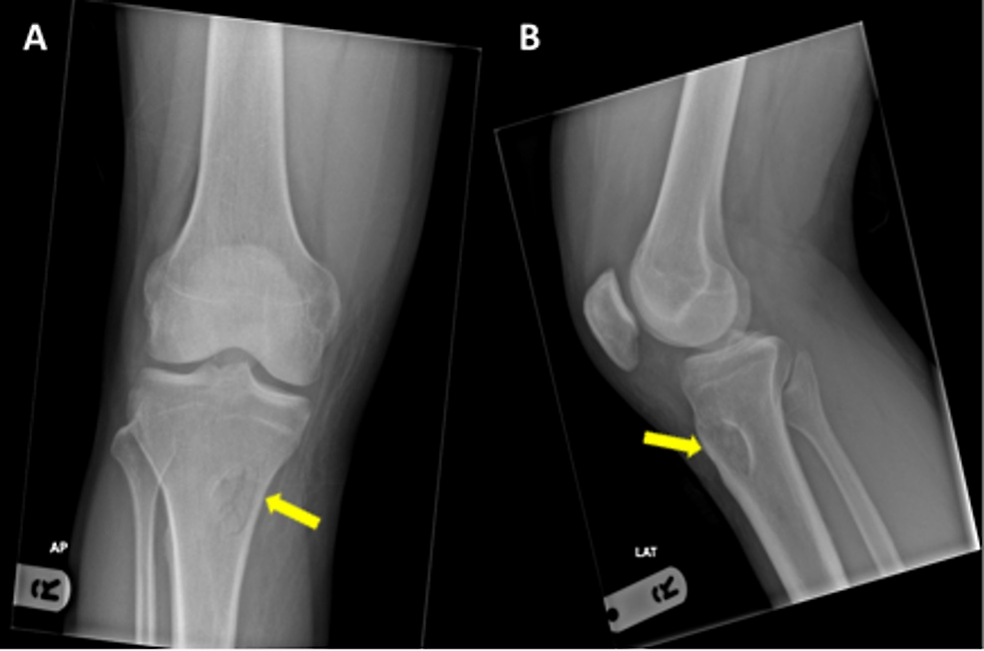
Radiographs of right tibia and fibula showing a well-circumscribed ovoid lucency with bony densities within in the anteromedial aspect of the right proximal tibia (yellow arrows).

Radiographs of tibia and fibula were obtained which showed an area of well-circumscribed ovoid lucency with bony densities within the anteromedial aspect of the right proximal tibia, which were suspected to be related to trauma or a bony lesion. No obvious overlying soft tissue swelling was seen and overlying fat planes were maintained (Figure 1).

**Figure 2:**
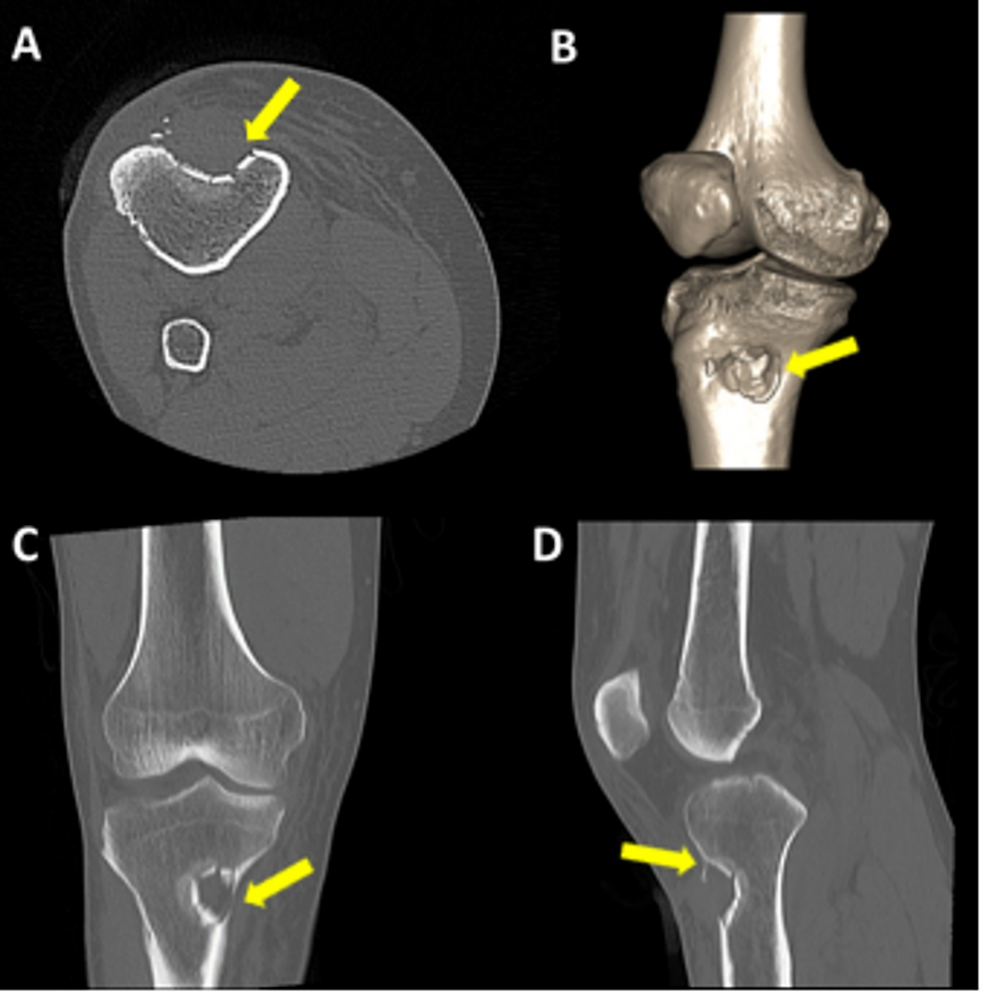
CT of right knee (A) axial; (B) coronal 3D reconstructed (C) coronal, and (D) sagittal reformatted images The bone window shows a focal depressed fracture involving the anteromedial cortex of the right proximal tibia (yellow arrows).

CT scan of the right knee was performed which demonstrated a focal depressed fracture involving the anteromedial cortex of the right proximal tibia. It measured approximately 29 mm in diameter with a maximum depression of about 15 mm. Bone fragments were noted along the depressed bone cortex. A 28 x 19 mm soft tissue was noted filling the depression with thin bone fragments within and adjacent to it. The soft tissue was suspected to be an organized hematoma. Adjoining soft tissue contusion was also noted with overlying skin laceration. No underlying bone neoplasm was evident (Figure 2) and further evaluation with magnetic resonance imaging (MRI) was recommended.

The patient was admitted to the orthopedics ward and under all aseptic precautions, the lacerated wound was sutured using Ethilon 3/0 sutures.

**Figure 3:**
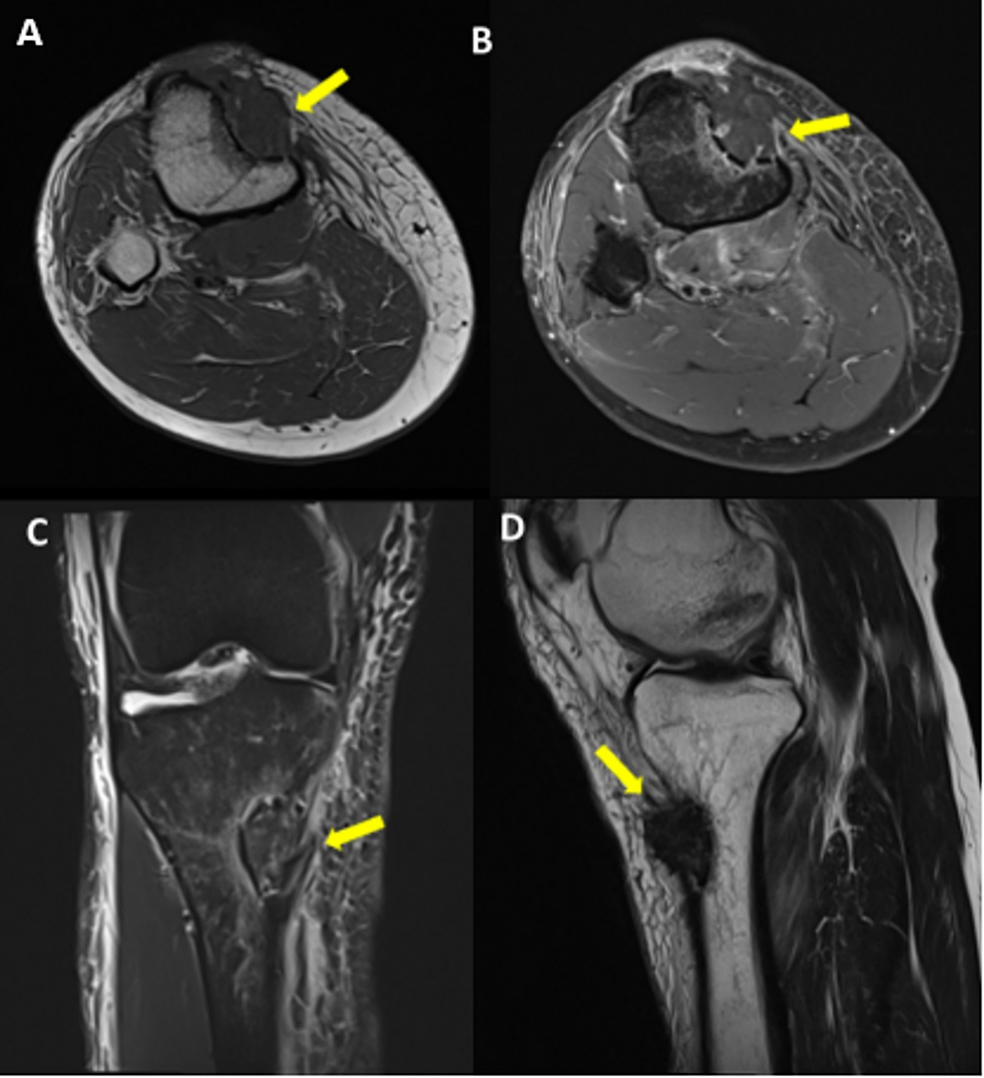
MRI of right knee T1 axial (A) pre contrast and (B) post contrast (C) T2 STIR coronal and (D) T2 sagittal The figures above show a hypointense lesion filling the depression fracture of the anteromedial cortex of the right proximal tibia (yellow arrows).

MRI of the knee was done which showed normal alignment of the knee joint and mild knee joint effusion. A focal depressed fracture was noted involving the anteromedial cortex of the right proximal tibia with bone marrow edema involving the adjoining subcortical bone. A focal soft tissue lesion that is hypointense on both T1 and T2-weighted images (suggesting an organized hematoma or fibrosis) is seen, filling the depression caused by the compression fracture. This lesion showed no significant enhancement other than a few small linear enhancing areas within it. No periosteal reaction is noted around the lesion or in the vicinity of the fractured cortex and no underlying bone neoplasm or pre-existing bone pathology is evident (Figure 3).

The patient was given oral analgesics and amoxicillin/clavulanate, 625mg for seven days. He improved symptomatically and was discharged home with axillary crutches for non-weight bearing, with orthopedics outpatient follow up. Follow-up in the orthopedics outpatient department after one month showed significant symptomatic improvement and follow-up CT scan at the same time showed stable changes.

## Discussion

Depression fractures are common in the tibial plateau and are caused by high-energy trauma. The most frequent mechanism is the hyperextension of the knee which leads the femoral condyle to cause axial compression on the anterior tibial plateau [1]. But to date, no case of depressed fracture of the metadiaphysis of tibia without the involvement of the articular surface has been reported. To our knowledge, this is the first case that describes this type of fracture in this location. The occurrence due to severe trauma without any preexisting underlying bone pathology in an adult is unprecedented.

The tibia is the main bone of the leg skeleton and the second largest bone in the body after the femur. Its sturdy structure gives it the ability to bear the body’s weight. It is also crucial in the stability of the knee and the ankle, and fractures of its metadiaphysis or proximal part can impair the steadiness and mobility of the knee. The tibial epiphysis articulates to the femur via the medial and lateral condyles that are separated by the intercondylar eminences along with the intercondylar tubercles. The metaphysis is a sensitive region as it is composed of cancellous low-density bone covered with a small cortical shell [2]. The shaft is roughly prismatic and formed of higher-density bone. With age, the microstructure of the tibial trabeculae changes depending on the load application patterns. This architectural anisotropy takes place with time and is accompanied by a significant decrease in bone density, with a thinning of trabeculae, which results in the transformation of the microstructure of the tibia, from a plate-like structure to a rod-like one. These changes occur physiologically after 60 years of age [3]. 

There are many classifications for proximal tibial fractures. The Orthopaedic Trauma Association (OTA) classification is widely used and contains 3 groups depending on the nature of the fracture: Group A encompasses the extraarticular fractures, group B the partially articular injuries, and group C the complete articular lesions. They are then subdivided into smaller groups depending on the pattern of the fracture lines [4]. The case that we described is an extraarticular fracture but cannot be put under any of the cited subcategories according to the 2018 OTA. 

There is a controversy regarding the definition of pathological fractures [5]. Three different entities look alike and amalgamate pathological fractures, stress fractures, and spontaneous fractures [6]. Pathological fractures refer to lesions that occur on an altered bony tissue, regardless of the type of the underlying condition (whether tumoral, acquired, or congenital), and regardless of the mechanism (whether it is high or low-intensity trauma). Stress fractures on the other hand occur in a normal bone that has been subjected to special mechanical conditions. Finally, spontaneous fractures happen when there is no impact or low impact; which means they are not always pathological, as they can occur on a seemingly normal bone [7]. No underlying pre-existing bone pathology is evident in our case on extensive imaging studies performed. The soft tissue filling the depression caused by the fracture in our case is likely a sequela of injury (organized hematoma). The symptoms of our patient were no different from any other tibial shaft fracture, namely excessive pain on palpation of the crackling line, which was associated with the inability to walk. 

Adequate imaging is key to direct proper management. Radiographs are usually the first imaging modality used in the context of an emergency. They can show the fracture line and enable the study of the underlying tissue, to a certain extent. However, in the context of pathological fractures, radiographs are insufficient to plan adequate interventions. CT scan can give a more precise analysis of the cortex, periosteum, and trabeculae [8]. MRI is known to be better for studying soft tissue and bone marrow. It allows a clearer visualization of the underlying structures, including the medullary cavity that might contain skip metastases. Ultra-high field MRI scanners with musculoskeletal (MSK) applications are available (although not generalized yet). They have demonstrated accuracy in the study of microarchitectural bone alterations, especially for osteoporosis, and are considered as a non-invasive alternative to DXA and QCT for studying bone density. 7T MRI has even shown the ability to provide data about the mineral content of the bones which was until now non-feasible using radiographic techniques [9]. To confirm an etiology a biopsy is necessary in most cases of pathological fractures. Nevertheless, when there is a lytic bone lesion in the context of known cancer with multiple metastases it is not needed [6].

Fractures of the upper part of the tibia are particularly difficult to manage because of several anatomical characteristics. First, the tibia is a load-bearing bone that creates an obstacle to consolidation and can cause malalignment. Plus, the anterior border of the shaft is not covered with adequate soft tissue which reduces blood supply and slows healing. Overall, the treatment strategy varies depending on the type of fracture and the underlying lesion, but it is recommended to go with the most conservative option that will allow a good reduction [2]. In our case, conservative orthopedic management was opted for, along with oral antibiotics and analgesics.

## Conclusions

Isolated focal depressed fracture of the proximal tibial metaphysis is a rare finding with no such described cases in the literature. X-rays form the primary investigative modality that can confirm the presence of a fracture. CT and MRI scans reveal a much more detailed image and a clearer relationship between the fracture and the surrounding structures besides excluding or confirming an underlying pre-existing bone pathology. These imaging modalities hold excellent sensitivity and specificity for fractures. Observation and conservative management are the mainstream treatment options. Patients with larger and debilitating fractures can opt for orthopedic surgical management.
